# Emerging Role of Linker Histone Variant H1x as a Biomarker with Prognostic Value in Astrocytic Gliomas. A Multivariate Analysis including Trimethylation of H3K9 and H4K20

**DOI:** 10.1371/journal.pone.0115101

**Published:** 2015-01-20

**Authors:** Athanasia Sepsa, Georgia Levidou, Antonis Gargalionis, Christos Adamopoulos, Anastasia Spyropoulou, Georgia Dalagiorgou, Irene Thymara, Efstathios Boviatsis, Marios S. Themistocleous, Kalliopi Petraki, George Vrettakos, Vassilis Samaras, Athanassios Zisakis, Efstratios Patsouris, Christina Piperi, Penelope Korkolopoulou

**Affiliations:** 1 First Department of Pathology, Laikon General Hospital, Athens University Medical School, Athens 115 27, Greece; 2 Department of Biological Chemistry, Athens University Medical School, Athens 115 27, Greece; 3 Department of Neurosurgery, Medical School, National and Kapodistrian University of Athens, Evangelismos Hospital, Athens 106 76, Greece; 4 Department of Pathology, Metropolitan Hospital, Athens 185 47, Greece; 5 Department of Neurosurgery, Metropolitan Hospital, Athens 185 47, Greece; 6 Department of Pathology, Red Cross Hospital, Athens 115 26, Greece; 7 Department of Neurosurgery, Red Cross Hospital, Athens 115 26, Greece; Peking University Health Science Center, CHINA

## Abstract

Although epigenetic alterations play an essential role in gliomagenesis, the relevance of aberrant histone modifications and the respective enzymes has not been clarified. Experimental data implicates histone H3 lysine (K) methyltransferases SETDB1 and SUV39H1 into glioma pathobiology, whereas linker histone variant H1.0 and H4K20me3 reportedly affect prognosis. We investigated the expression of H3K9me3 and its methyltransferases along with H4K20me3 and H1x in 101 astrocytic tumors with regard to clinicopathological characteristics and survival. The effect of SUV39H1 inhibition by chaetocin on the proliferation, colony formation and migration of T98G cells was also examined. SETDB1 and cytoplasmic SUV39H1 levels increased from normal brain through low-grade to high-grade tumors, nuclear SUV39H1 correlating inversely with grade. H3K9me3 immunoreactivity was higher in normal brain showing no association with grade, whereas H1x and H4K20me3 expression was higher in grade 2 than in normal brain or high grades. These expression patterns of H1x, H4K20me3 and H3K9me3 were verified by Western immunoblotting. Chaetocin treatment significantly reduced proliferation, clonogenic potential and migratory ability of T98G cells. H1x was an independent favorable prognosticator in glioblastomas, this effect being validated in an independent set of 66 patients. Diminished nuclear SUV39H1 expression adversely affected survival in univariate analysis. In conclusion, H4K20me3 and H3K9 methyltransferases are differentially implicated in astroglial tumor progression. Deregulation of H1x emerges as a prognostic biomarker.

## Introduction

Gliomas are highly invasive and vascularised neoplasms accounting for more than 70% of all brain tumors. Despite recent advances in surgery, radiotherapy and chemotherapy, survival of high-grade glioma patients remains poor [1]. The lack of robust treatment options has propelled a search for markers that could identify subgroups of patients likely to benefit from molecularly targeted therapies. An interplay between genetic and epigenetic mechanisms has been proposed to underline glioma pathogenesis and progression [2].

Epigenetic modifications, in contrast to genetic alterations, are defined as heritable changes in gene expression that do not result in any alteration of DNA sequence. Their mechanisms include DNA methylation, covalent histone modification, dynamic shuffling of histone variants and microRNAs [3]. Histones are proteins closely associated with DNA molecules [4]. Research during the last decade has revealed that the role of histones goes beyond the maintenance of DNA structure to the regulation of gene expression by virtue of altering DNA compaction [5]. In humans, five histone families have been identified-H1, H2A, H2B, H3 and H4 [4]. The H1 family represents the linker histones which bind to the linker DNA between the nucleosome cores [6, 7]. This family includes 11 subtypes of lysine-rich proteins functioning to ensure genomic integrity and regulating the transcription of genes involved in aging, DNA repair, DNA methylation, imprinting and apoptosis [8, 9]. H3 and H4 together with H2A and H2B belong to the core histone group and form octamers around which DNA is wrapped (nucleosome core particle) [10]. Posttranslational modifications of N-terminal tail of these histones (HPTMs) constitute the histone code with profound impact on chromatin folding contributing to regulation of gene expression [11]. Among the several types of covalent modifications of histone amino acid tails, such as acetylation, methylation, phosphorylation, ubiquitination, and SUMOylation, lysine (K) methylation is the most prominent with ability to activate or suppress transcription depending on the histone residue that is methylated [12]. In particular, trimethylation of H3K9 and H4K20 is associated with silencing of transcription by promoting the interaction of the modified histones with heterochromatin protein 1 [10, 13, 14]. Histone lysine methylation is regulated by histone lysine methyltransferases (HKMTs) and demethylases (HKDMs) [2]. HKMTs catalyzing the H3K9me3 (trimethylation of H3K9) include the SET domain family of HKMTs, SETDB1 and SUV39H1, as well as G9A.

SETDB1 (SET domain bifurcated 1) is the only methyltransferase of euchromatin origin that can also mediate H3K9 trimethylation. It is tightly linked to DNA methylation [15] and its proper function is considered essential for early mouse brain development [16]. Furthermore, the best characterized SUV39H1 and its homologue SUV39H2 are required for heterochromatin formation. SUV39H1-mediated H3K9 methylation has been linked to silencing of the tumor suppressor genes in acute myeloid leukemia, whereas its default function is to maintain genome stability by limiting the acute activation of oncogenes [17]. Deregulation of SUV39H1 has been associated with oncogenic phenotype in several human malignancies [2]. The reversible nature of histone methylation by chemical inhibitors or demethylating enzymes represents an appealing therapeutic approach for several major diseases including cancer. The specific inhibitor of SUV39H1, chaetocin has been reported to reduce H3K9 methylation, impose oxidative stress and serve as potent anti-myeloma agent in vitro and in vivo [18].

To date there is limited evidence regarding the role of methyltransferases and histone lysine methylation in the pathogenesis of astrocytic tumors. In a recent study of 284 gliomas, trimethylation of H3K9 was found in all grades of astrocytic tumors [12]. Furthermore, Spyropoulou et al. [19] found increased expression of SETDB1 and SUV39H1 in fifteen glioma tissues compared with normal brain. The potential significance of linker histone variant H1.0 has only recently been proposed based on the findings of a small series of astrocytoma patients [20]. The paucity of information in this regard prompted us to undertake an immunohistochemical analysis of H1x, H3K9me3, H4K20me3, SETDB1 and SUV39H1 in a large series of astrocytic tumors in order to investigate the interrelations between the expression levels of these molecules and their potential importance in glioma progression. Validation of immunohistochemistry for H3K9me3, H4K20me3 and H1x was performed by Western immunoblotting in normal brain and glioma tissue, as well as in astroglial cell line SVG p12 and glioblastoma cell line T98G. The effect of SUV39H1 inhibition by chaetocin on the proliferation, colony formation and migration of T98G cells was also examined. Furthermore, analysis of these molecules with respect to clinicopathological parameters and patients’ survival was also performed.

## Materials and Methods

### Patients’ description

This is a study of 101 adult patients with supratentorial diffuse infiltrating astrocytomas (grades 2 to 4) for which archival primary tumor material at diagnosis, prior to radio/chemotherapy, was available. Patients had been diagnosed in the First Department of Pathology, Laikon Hospital, National and Kapodistrian University of Athens, and treated as well as followed-up in Evangelismos, Asklepeion and Metropolitan Hospitals between 2003 and 2009. In all cases, the diagnoses and grading were peer-reviewed according to the principles laid down in the latest World Health Organization WHO Classification [21]. Distinction between primary (59 cases) and secondary (19 cases) glioblastomas was based on WHO criteria and IDH1-R132H expression [21]. Written informed consent was obtained from all patients and the study was approved by the University of Athens Medical School Ethics Committee (17/2/2012, protocol number 5105). Follow-up information was available in 99 patients. Postoperative radiation consisted of a total dose of 60 Gy in 30 to 33 fractions. According to the existing treatment protocols, no chemotherapy was administered for cases diagnosed before 2007. Table 1 summarizes the demographic data of our patients.

**Table 1:** Demographic data of patients’ and validation cohort.

**Variable**	**Patients’ cohort (n = 101)**	**Validation’s cohort (n = 66)**
	**Median (range)**	**Median (range)**
**Age**	59 (19–84) years	59 (25–77)
	**Number of cases (%)**	**Number of cases (%)**
**Gender:**		
Male	58 (57.1%)	23 (34.8%)
Female	43 (42.6%)	43 (65.2%)
**Grade:**		
2	11 (10.9%)	13(19.7%)
3	12 (11.9%)	0 (0%)
4	78 (77.2%)	53 (80.3%)
**Events:**		
Death	74 follow-up period: median 9 (range 1–39) months	45 follow-up period: median 9 (range 1–86) months
Censored	25 follow-up period: median 18 (range 3–48) months	21 follow-up period: median 11 (range 0.5–36) months
Lost to follow-up	2	-
**Surgery:**		
Partial	31 (30.7%)	24 (36.4%)
Complete	66 (65.3%)	41 (62.1%)
NA	4 (4%)	1 (1.5%)
**Radiotherapy:**		
Yes*	12 (11.9%)	31 (47%)
No	78 (77.2%)	5 (7.6%)
NA	11 (10.9%)	30 (45.4%)
**Chemotherapy (Temozolomide):**		
Yes	39 (38.6%)	44 (66.7%)
No	14 (13.9%)	0 (0%)
NA	48 (47.5%)	22 (33.3%)

NA = not available

* postoperative radiotherapy (a total dose of 60 Gy in 30 to 33 fractions)

### Culture of Glioma Cell Line and chaetocin treatment

Human astroglial SVG p12 cell line (CRL-8621) was obtained from ATCC and human Caucasian glioblastoma cell line T98G (Cat. No: 92090213) was obtained from ECACC. Both cell lines were kindly provided by Robert W. Lea, Department of Biological Sciences, University of Central Lancashire, Preston, UK [2]. SVG p12 cells were cultured in Eagle’s Minimum Essential Medium (Gibco, Life Technologies) supplemented with 10% fetal bovine serum-FBS (Gibco, Life Technologies): 1% penicillin-streptomycin mixture (10,000 U/ml of penicillin and 10,000 µg/ml of streptomycin Gibco, Life Technologies): and 0.1% Fungizone antimycotic (250 μg/ml Gibco, Life Technologies). Glioma cells were cultured in RPMI 1640 medium GlutaMAX (Gibco, Life Technologies) supplemented with 10% fetal bovine serum-FBS (Gibco, Life Technologies): 1% penicillin-streptomycin mixture (10,000 U/ml of penicillin and 10,000 µg/ml of streptomycin Gibco, Life Technologies): and 0.1% Fungizone antimycotic (250 μg/ml Gibco, Life Technologies). Cell cultures were maintained at 37°C in a humidified atmosphere containing 5%CO_2_ -95%air.

For inhibition of SUV39H1 activity, T98G cells were cultured in twelve-well plates and upon 70–80% confluence they were incubated in the presence or absent of 200 nM and 400 nM of chaetocin (Enzo lifesciences, GR-349–0200 reconstituted in DMSO) for 12 and 24 hr at 37°C in 5% CO_2_ humidified air. Upon completion of time points, cells were selected for protein extraction.

### Immunohistochemical staining

Immunostaining was performed on paraffin-embedded 4 μm sections of formalin fixed tumor tissue. Briefly, following deparaffinization and rehydration, sections were treated in 3% H_2_O_2_ for 30 min to quench endogenous peroxidase and then with blocking serum. Antigen retrieval was performed in a microwave oven at 750W and then the primary antibodies were applied overnight at 4°C. Details regarding the retrieval methods as well as the primary antibodies used (source, dilutions) are listed in Table 2. Following this step, the two-step peroxidase conjugated polymer technique was applied (DAKO invision kit K5007, DAKO, Carpinteria, CA) for 30 min. For visualization of immunoreactivity 3,3΄-diaminobenzidine (DAB) was used as a substrate until the desired signal intensity developed. Counterstaining was performed with haematoxylin. Negative controls (i.e sections in which the primary antibody was substituted with non-immune serum) were also stained in each run. Immunohistochemical evaluation was performed by three pathologists (PK, GL, AS): without knowledge of the clinical information. Nuclear and cytoplasmic immunoreactivity was recorded separately. A Histo-score (H-score) based on the percentage of stained neoplastic cells (labelling index-LI) multiplied by staining intensity was calculated.

**Table 2:** Characteristics of primary antibodies used in immunohistochemical (IHC) analysis.

**Protein**	**Clone**	**Company**	**Catalog no.**	**Raised in**	**Positive controls**	**No of stained slides for IHC**	**Antigen retrieval method for IHC**	**Dilution and incubation time**
H1x	polyclonal	Abcam, Cambridge, UK	Ab 31972	rabbit	Human breast carcinoma	98	Citrate buffer pH = 6	1:500, overnight (4°C)
H3K9me3 (trimethylated at Lys9)	monoclonal	Millipore, Massachusetts, USA	CMA 308	mouse	Human breast carcinoma	99	EDTA pH = 9	1:100, overnight (4°C)
H4K20me3 (trimethylated at Lys20)	monoclonal	Abcam, Cambridge, UK	Ab 78517	mouse	Human breast carcinoma and normal human skin	99	Citrate buffer pH = 6	1:500, overnight (4°C)
SETDB1	polyclonal	Sigma-Aldrich chemie GmbH, Steinheim, Gernany	HPA 018142	rabbit	Normal human colon	98	EDTA pH = 9	1:100, overnight (4°C)
SUV39H1	polyclonal	Novus Biologicals, Colorado, USA	NBP1 21367	rabbit	Human breast carcinoma	100	Citrate buffer pH = 6	1:200, overnight (4°C)
IDH1-R132H	monoclonal	Dianova, Hamburg, Germany	Clone H09	mouse	Low-grade astrocytoma	101	Citrate buffer, pH = 6	1:50, overnight (4°C)

### Western immunoblotting analysis

Protein extraction from normal brain (3 samples) and glioma tissues of different grade (3 samples from grades II and IV respectively), astroglial cell line SVG p12 and glioma cell line T98G was performed using ice-cold RIPA buffer (Thermo Scientific, Rockford, IL, USA) and a protease inhibitor cocktail (Thermo Scientific). Bradford assay (Bio-Rad) was used to assess protein concentration in the extracts. Proteins were separated by SDS-polyacrylamide gel electrophoresis and transferred to a nitrocellulose membrane (Porablot NCP, Macherey-Nagel). Membranes were blocked for 1h in room temperature in Phosphate Buffered Saline Tween-20 (PBST) with 5% non-fat milk. Subsequently, membranes were incubated overnight at 4°C with the following primary antibodies: anti-H1x, anti-H3K9me3, anti-H4K20me3 or anti-Actin (MAB-1501, Millipore, Bedford, MA, USA). Antibodies against H1x, H3K9me3 and H4K20me3 were diluted 1:500 in PBST containing 1% non-fat milk whereas the anti-Actin antibody was diluted 1:5000 in the same buffer. The membranes were then incubated with the HRP—conjugated secondary antibodies for 1h in room temperature. Secondary antibodies goat anti-rabbit IgG-HRP (sc-2004, Santa Cruz Biotechnology) and goat anti-mouse IgG-HRP (sc-2005, Santa Cruz Biotechnology) diluted 1:2500 were used. The detection of the immunoreactive bands was performed with the SuperSignal WestPico Chemiluminescent HRP Substrate kit (Thermo Scientific).

Relative protein amounts were evaluated by a densitometric analysis using Image J software (La Jolla, CA, USA) and normalized to the corresponding Actin levels. All experiments have been performed at least 3 times and representative results of one experiment are shown.

### Cell Proliferation Assay

The assessment of T98G cell proliferation in the presence or absence of chaetocin was performed with the 3-(4,5-dimethylthiazol-2-yl)-2,5-diphenyl-tetrazolium bromide (MTT) assay. 12 h and 24 h post treatment, T98G cells were collected and plated into 96-well in triplicate where left overnight. The next day, the medium was replaced with MTT (Sigma-Aldrich, Athens, Greece) diluted in serum-free, phenol red (PR)-free medium at a final concentration of 1 mg/ml, and cells were incubated another 3h at 37°C in a 5% CO_2_ atmosphere. After incubation with the MTT reagent, the MTT-formazan product was solubilized thoroughly in isopropanol and the absorbance was measured at 570 nm with a background wave length of 690 nm. Each experiment was conducted in triplicate.

### Clonogenic assay

T98G cells treated with chaetocin (200nM and 400 nM) for 12h were seeded in 12-well plates at a density of approximately 500 cells per well and left to form colonies for 10–15 days. Cells were then fixed with a solution containing 25% acetic acid and75% methanol and stained with hematoxylin for 20min. Individual colonies were counted using an inverted microscope and precise electronic counter.

### Migration Assay

T98G cells were seeded in 12-well plates (at a density of 10 × 10^4^ cells per well) and maintained in a humidified atmosphere of 5% CO_2_–95% air at 37°C. At 20h after chaetocin treatment (200nM and 400nM), the cell monolayer was scraped with a sterile 200-μL pipette tip (marking the point of zero migration); fresh medium was added to the plates which were further returned to the incubator for 20h. After completion of 20h incubation, the samples were washed twice very gently with PBS, pH 7.2. Each well was photographed at ×4 and ×20 magnifications using computer-assisted microscopy. Phase-contrast images were taken at the beginning (0h) and after 20h of incubation for the same scratch area. The pictures were analyzed with the WimScratch software (Wimasis image analysis platform). The results were expressed as percentages of scratched and cell-covered areas.

### Statistical Analysis

Statistical analysis was performed by a M.Sc. Biostatistician (GL). In the basic statistical analysis H1x, SETDB1, H4K20me3, H3K9me3 and SUV39H1 expression were treated as continuous variables. Associations of the molecules under study with clinicopathological parameters were tested using non-parametric tests with correction for multiple comparisons (Kruskal-Wallis ANOVA, Mann-Whitney U-test, Fisher’s exact test and Spearman’s rank correlation coefficient, as appropriate).

Survival analysis was performed using death by disease as endpoint. The effect of various clinicopathological parameters (age, sex, radiotherapy, chemotherapy, extent of surgical resection and histological grade) as well as H1x, SETDB1, H4K20me3, H3K9me3 and SUV39H1 immunoreactivity on clinical outcome was assessed by plotting survival curves according to the Kaplan-Meier method and comparing groups using the log-rank test. Numerical variables were categorized on the basis of cut-off values provided by ROC curves. Multivariate survival analysis using Cox’s proportional hazard estimation model was performed for those parameters that were proven to be significant in univariate analysis in order to evaluate the predictive power of each parameter independently of the others. Due to the increased number of missing values, chemotherapy was excluded from multivariate analysis in the entire cohort but its potential effect with regard to the significant parameters arising from multivariate analysis was tested in the subgroup of patients for which this information was available. To avoid any “data-driven” categorization numerical variables (i.e. the H-scores of the examined proteins) were entered in multivariate analysis in continuous form.

Statistical calculations were performed using the statistical package STATA 11.0 for Windows. Power calculation was performed using NCSS 8.0 for Windows. All results with a two-sided p level ≤0.05 were considered statistically significant.

### Validation cohort

An independent set of patients with astrocytic gliomas was used to validate the chosen cut-off values for the expression of H1x in univariate analysis. The results of univariate survival analysis for H1x expression in the population group were used to calculate the required number of patients in the validation group for an adequately powered analysis (80%). In order to detect a difference of 0.29410 between 0.11250 and 0.40660, that is the proportion of patients surviving in the low- and high- expressor group concerning H1x H-score, as calculated in the population cohort- using a two-sided log-rank test, and to achieve 80% power at a 0.05 significance level, 38 patients would be needed [22]. Using the same method in the group of glioblastomas (to detect a difference of 0.32560 between 0.71660 and 0.39100 as observed in the patient’s group) 48 patients would be needed [22]. The validation group we used consisted of 66 patients (53 patients with glioblastomas and 13 with diffuse astrocytomas), originally diagnosed and treated at Red-Cross Hospital between 2007 and 2011.The demographic data of this cohort are shown in Table 1.

## Results

### H1x, H4K20me3, H3K9me3, SETDB1, and SUV39H1 expression in astrocytic tumors and normal brain tissue (Table 3)

**Table 3:** Distibution of H1x, SETDB1, H4K20me3, H3K9me3 and SUV39H1 H-score according to histological grade.

	**H1x**	**H4K20me3**	**H3K9me3**	**SETDB1**	**SUV39H1**	
					**Nuclear**	**Cytoplasmic**
	**Median (range)**					
**Total**	85 (0–300)	40 (0–300)	60 (0–300)	98.75 (0–294)	2 (0–140)	30 (0–285)
**Histological grade**						
2	160 (52.5–300)	165 (6–300)	100 (0–300)	100 (5–270)	20 (0–140)	4 (0–110)
3	55 (2–120)	64.5 (4.5–270)	52.5 (2–225)	57.5 (5–245)	12.5 (0–90)	5 (0–180)
4	75 (0–297)	30 (0–285)	60 (0–285)	105 (2–294)	0 (0–30)	60 (0–285)

**Figure 1:**
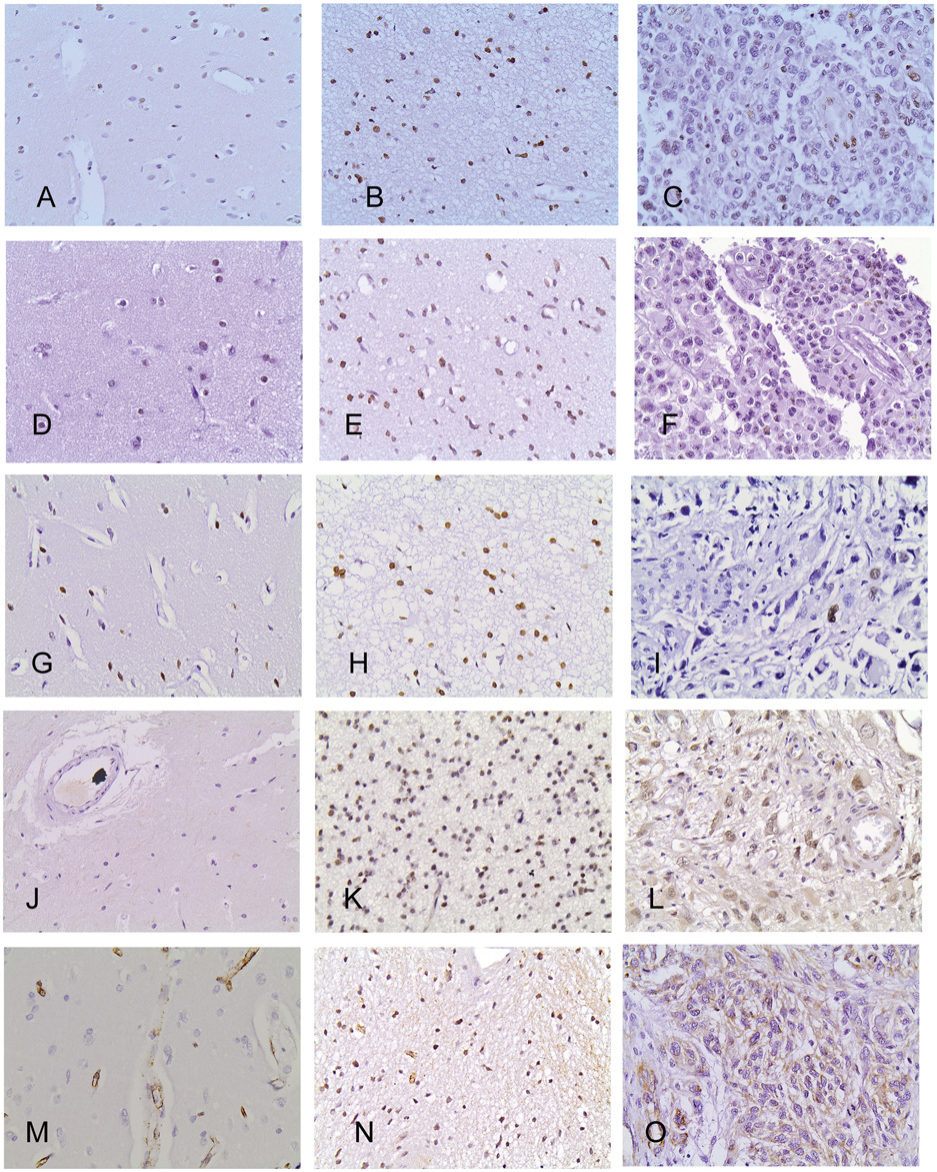
Immunohistochemical expression of H1x (A, B, C): H4K20me3 (D, E, F): H3K9me3 (G, H, I): SETDB1 (J, K, L): and SUV39H1 (M, N, O) in normal brain tissue (A, D, G, J, M): grade 2 atrocytomas (B, E, H, K, N) and glioblastomas (C, F, I, L, O). Normal brain tissue displays lower SETDB1, cytoplasmic or nuclear SUV39H1 but higher H3K9me3 immunoreactivity than neoplastic tissue. Within astrocytic tumors, H1x, H4K20me3 and nuclear SUV39H1 immunoreactivity decrease from grade 2 through grade 3 to grade 4. On the contrary, SETDB1 and cytoplasmic SUV39H1 expression is higher in glioblastomas than in lower grades.

H1x immunoreactivity was nuclear and was observed in 97/98 (98%) of cases in the population cohort and in all cases of the validation cohort, with an H-score ranging from 0.5 to 300 (Fig. 1B,C). The immunoreactivity showed a predilection for perinecrotic areas, whereas cells in mitosis showed a cytoplasmic staining pattern. Endothelial cells were always positive and served as internal positive controls for each case. H4K20me3 and H3K9me3 nuclear immunoreactivity was seen in 99% (94/99) and 94% (94/100) of the examined cases, with an H-score ranging from 0.5–300 and 2–300 respectively (Fig. 1 E, F, H, I). Endothelial cells were always positive for H3K9me3 and H4K20me3, whereas cells in mitosis were consistently negative.

SETDB1 nuclear immunoreactivity was recorded in 98% of the cases with an H-score ranging from 1 to 294 (Fig. 1 K, L). Only 14/98 examined cases (14.2%) displayed also cytoplasmic immunoexpression. Neurons as well as endothelial cells were positive for SETDB1 and served as internal positive controls in the negative cases.

SUV39H1 expression was nuclear in 56/100 and cytoplasmic in 87/100 of the examined cases (Fig. 1 N, O). Nuclear SUV39H1 H-score ranged from 1–140 whereas cytoplasmic one from 0.5–285. Most of the endothelial cells displayed cytoplasmic immunoreactivity.

**Figure 2:**
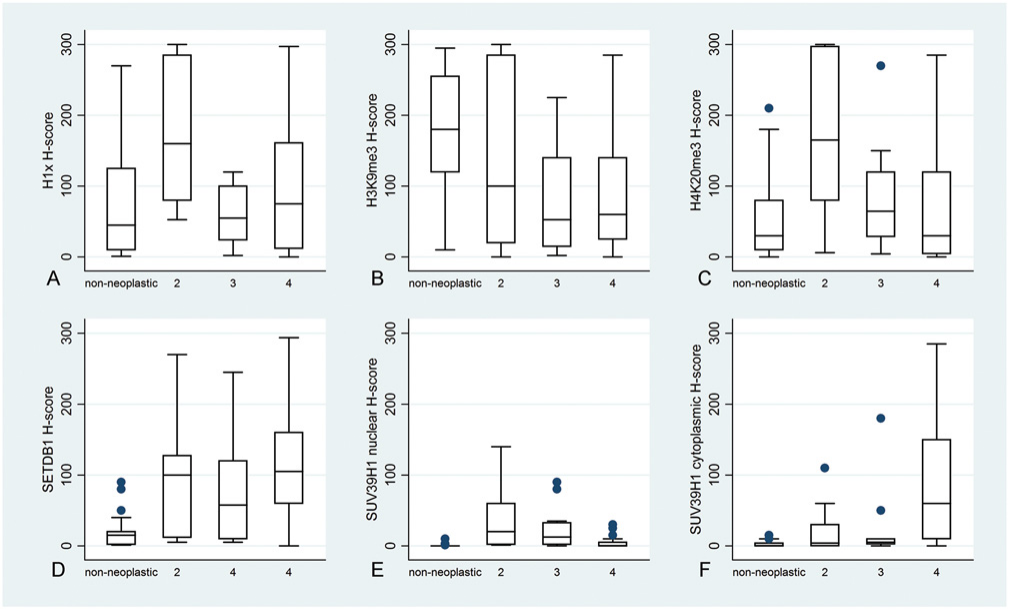
H1x (A): H4K20me3 (B): H3K9me3 (C): SETDB1 (D): and SUV39H1 nuclear (E) and cytoplasmic (F) H-score in normal brain tissue and in astrocytomas according to histological grade. H3K9me3 expression was higher in normal brain showing no association with grade, whereas H1x and H4K20me3 expression was significantly increased in grade 2 as compared to normal brain or high grades. H1x, H4K20me3 and nuclear SUV39H1 levels decrease with progression in grade, whereas SETDB1 and SUV39H1 are positively associated with grade.

Adjacent normal brain tissue displayed lower SETDB1, cytoplasmic and nuclear SUV39H1 and higher H3K9me3 H scores when compared to the neoplastic tissue (SETDB1–34 cases, p<0.0001 SUV39H1–32 cases, nuclear immunoexpression p = 0.0003 and cytoplasmic immunoexpression p<0.0001 and H3K9me3 26 cases, p<0.0001, Mann-Whitney U-test, Fig. 1 G, J, M, Fig. 2 C-F). With regard to H3K9me3, normal brain tissue immunoreactivity was significantly higher from grades 3 (p = 0.0039) and 4 (p = 0.0001) but did not differ from grade 2 (p>0.10). On the contrary, H1x and H4K20me3 immunoreactivity in the normal brain tissue did not significantly differ from that observed in grades 3 (p = 0.9882 and p = 0.2121 respectively) and grades 4 (p = 0.2846 and p = 0.8520 respectively) but was lower than that observed in grades 2 (p = 0.0013 and p = 0.0034 respectively, Fig. 1 A,D, Fig. 2 A,B).

**Figure 3:**
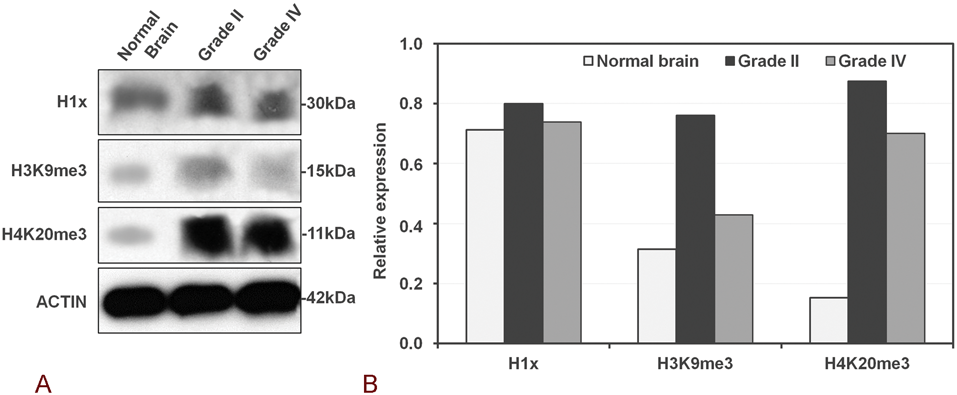
Western blot analysis of H1X, H3K9me3 and H4K20me3 in glioma tissues and normal brain. Representative figures of western blot analysis of H1X, H3K9me3 and H4K20me3 expression levels in glioma tissues of different grade and normal brain. Densitometric quantification of protein levels from one representative experiment (normalised to the actin levels) A trend of increased levels of H1x and H4K20me3 in grade 2 tumors is observed which however, especially regarding H1x is less prominent, probably due to the small number of samples analyzed by Western blot.

The expression patterns for H1x, H3K9me3 and H4K20me3 observed in the Western Blot analysis of total lysates from normal brain tissue as well as glioma tissues of various grades (Fig. 3), also showed a trend of increased levels of H1x and H4K20me3 in grade 2 tumors which however, especially regarding H1x was less prominent, probably due to the small number of samples analyzed by Western blot.

### Correlations among H1x, H4K20me3, H3K9me3, SETDB1, and SUV39H1 (Table 3)

H1x H-score was positively correlated with H4K20me3 (R = 0.3754, p = 0.0002) and H3K9me3 H-scores (R = 0.3417, p = 0.0007). These correlations remained significant after adjustment for histological grade. A positive correlation was also observed between H3K9me3 and SETDB1 (R = 0.2088, p = 0.0423) as well as a negative one between nuclear and cytoplasmic SUV39H1 expression (R = -0.4579, p<0.0001).

### Associations of H1x, H4K20me3, H3K9me3, SETDB1, and SUV39H1 with IDH1-R132H and clinicopathological features

A negative correlation emerged between H1x, H4K20me3 as well as nuclear SUV39H1 H-score and histological grade (Kruskal-Wallis ANOVA, p = 0.0184, p = 0.0064, p = 0.0022 respectively, Table 4, Fig. 2A,B,E). On the other hand, SETDB1 H-score and cytoplasmic SUV39H1 H-score showed a positive correlation with histological grade (Mann Whitney U test 2/3 vs 4 p = 0.0271 for the former relationship, Kruskal- Wallis ANOVA, 2 vs 3 vs 4, p = 0.0001 for the latter relationship, Table 4, Fig. 2A,D,F).

**Table 4:** Correlations among H1x, SETDB1, H3K4me3, H3K9me3 and SUV39H1 H-score in the entire cohort (Results of Spearman correlation coefficient). NS: not significant).

	**H1**	**H3K9me3**	**H3K4me3**	**SETDB1**	**SUV39H1 nuclear**
**H3K9me3**	R = 0.3754, p = 0.0002	NS			
**H3K4me3**	R = 0.3417, p = 0.0007	NS	NS		
**SETDB1**	NS	R = 0.2088, p = 0.0423	R=-0.1844, p = 0.0052		
**SUV39H1 nuclear**	R = 0.2188, p = 0.0332	p>0.10	R = 0.3059, p = 0.0026	NS	
**SUV39H1 cytoplasmic**	NS	NS	NS	NS	R = -0.4579, p<0.0001

### Survival analysis

**Figure 4:**
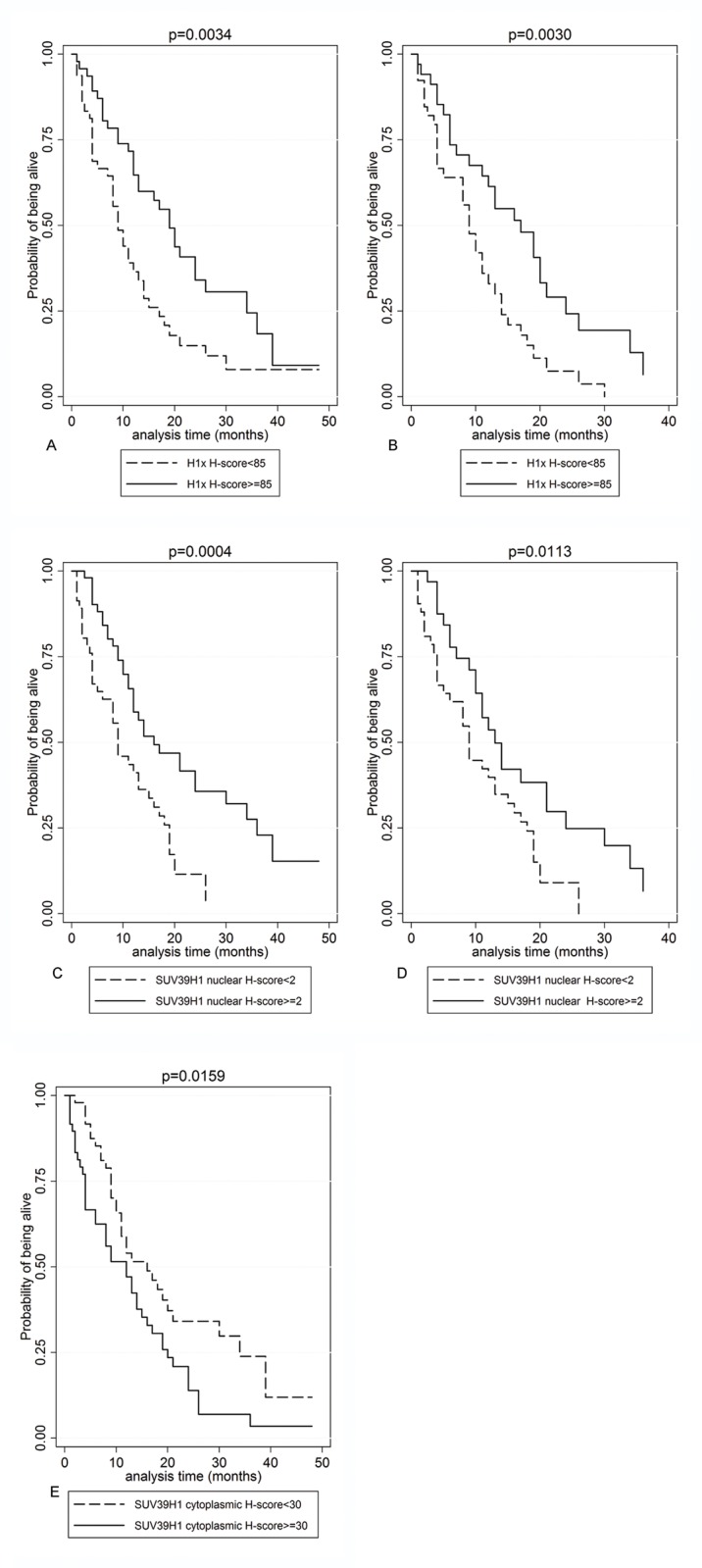
Kaplan-Meier survival curves according to H1x (A, B): nuclear SUV39H1 (C, D) and cytoplasmic SUV39H1 (E) in the entire cohort (A, B, C) as well as in glioblastomas (B, D) in the patients’ cohort. Increased H1x, increased nuclear SUV39H1 and decreased cytoplasmic SUV39H1 expression were associated with improved overall survival.

Univariate survival analysis was carried out in the entire cohort and in glioblastomas separately. The results are presented in Table 5. H1x and nuclear SUV39H1 expression were correlated with improved overall survival in the entire cohort (p = 0.0034 and p = 0.0004 respectively, Fig. 4A,C) as well as in glioblastomas (p = 0.0030 and p = 0.0113 respectively, Fig. 4B,D). Increased SETDB1 H-score was marginally correlated with favourable outcome in the entire cohort (p = 0.0662): whereas high cytoplasmic SUV39H1 H-score was correlated with adverse prognosis (p = 0.0159, Fig. 4E) in the entire cohort.

Multivariate survival analysis results, including all parameters for the patients for whom staining results for the molecules under study were available, are presented in Table 6. H1x expression emerged as an independent predictor of prognosis (HR = 0.995, p = 0.001): along with histological grade and patients’ age in the entire cohort. Importantly, H1x prognostic significance remained when analysis was restricted to glioblastomas (HR = 0,994, p = 0.001): as well as when it was adjusted for the administration of chemotherapy (HR = 0.993, p = 0.013).

**Table 5:** Results of univariate survival analysis (log-rank test) for overall survival.

	**Entire cohort**	**Glioblastomas**
Histological grade *(ΙI vs III vs IV)*	**<0.0001**	-
Age *(>35 vs< = 35 years)*	**0.0001**	**0.0089**
Gender *(1: male, 2: female)*	0.7425	0.7356
Surgical excision *(0: partial, 1: complete)*	0.4729	**0.0128**
Radiotherapy *(0: no, 1: yes)*	0.1094	**<0.0001**
Chemotherapy *(0: no, 1: yes)*	**0.0130**	**<0.0001**
H1x H-score *(<85 vs ≥85)*	**0.0034**	**0.0030**
H3K9me3 H-score *(<60 vs ≥60)*	0.1262	0.2558
H4K20me3 H-score *(<40 vs ≥40)*	0.5666	0.5705
SETDB1 H-score *(<98.75 vs ≥98.75)*	0.0662	0.2935
SUV39H1 nuclear H-score *(<2 vs ≥2)*	**0.0004**	**0.0113**
SUV39H1 cytoplasmic H-score *(<30 vs ≥30)*	**0.0159**	0.1512
IDH1-R132H (*positive vs negative)*	0.2891	0.3182

**Table 6:** Cox proportional Hazards model including all molecules under study in the entire cohort (n = 99 patients, model A), as well as in glioblastomas (n = 76, model B).

		**Hazard ratio (HR)**	**p-value**	**95% Confidence interval of HR**	
**A**	**H1x H-score**	0,995	**0,001**	0,993	0,998
	**SETDB1 H-score**	0,997	**0,026**	0,994	1,000
	**SUV39me3 nuclear H-score**	1,002	0,272	0,999	1,005
	**SUV39me3 cytoplasmic H-score**	1,006	0,481	0,990	1,022
	**Histological grade**	2,124	**0,032**	1,067	4,231
	**Patient’s age**	1,041	**0,002**	1,014	1,069
**B**	**H1x H-score**	0,445	**0,009**	0,242	0,817
	**SUVme3 nuclear H-score**	0,589	0,095	0,316	1,097
	**Patient’s age**	1,034	0,062	0,998	1,070
	**Radiotherapy**	0,204	**0,002**	0,076	0,548
	**Surgery**	0,428	**0,009**	0,227	0,808

### Survival analysis-Validation group

**Figure 5:**
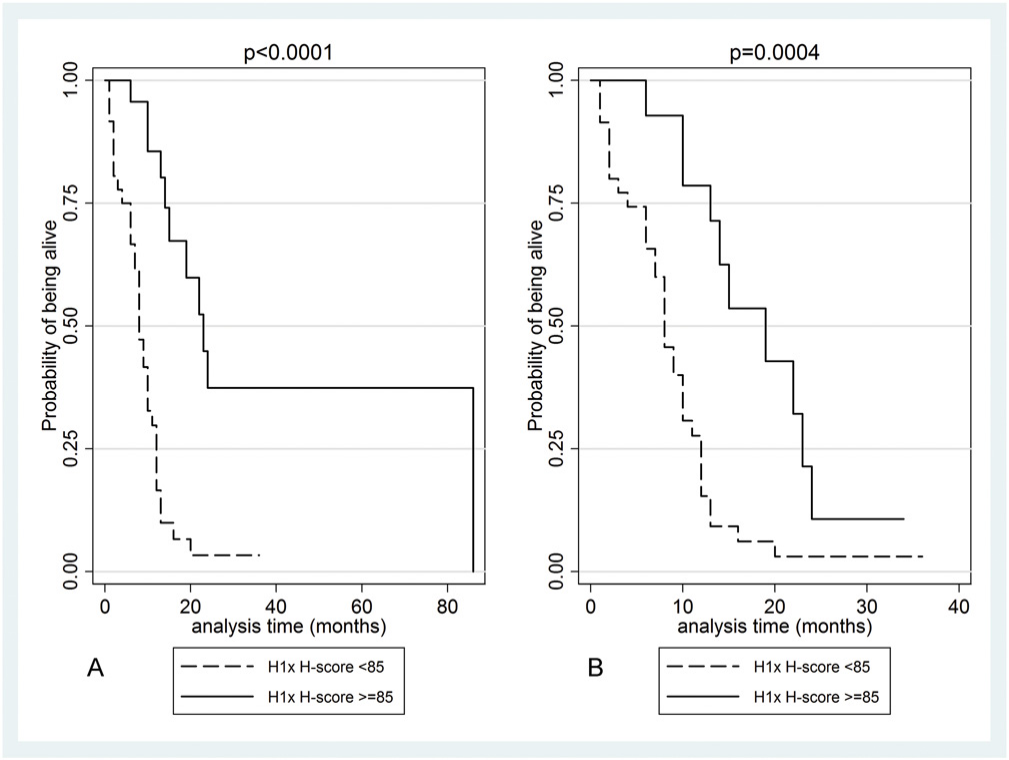
Kaplan-Meier survival curves according to H1x in the entire cohort (A) as well as in glioblastomas (B) in the validation cohort. Increased H1x expression implied a higher probability of survival.

The overall survival was significantly lower in the H1x low-expressor compared to the H1x high-expressor group in the entire cohort (log-rank test, p<0.0001, Fig. 5A) as well as in glioblastomas (log rank test, p = 0.0004, Fig. 5B). In a multivariate survival model in glioblastomas (including patients’ age, radiotherapy and chemotherapy) H1x H-score retained its prognostic significance (HR = 0.258, p = 0.016), along with patients age.

### Suppression of SUV39H1 activity results in reduced cell proliferation of glioma cells

**Figure 6:**
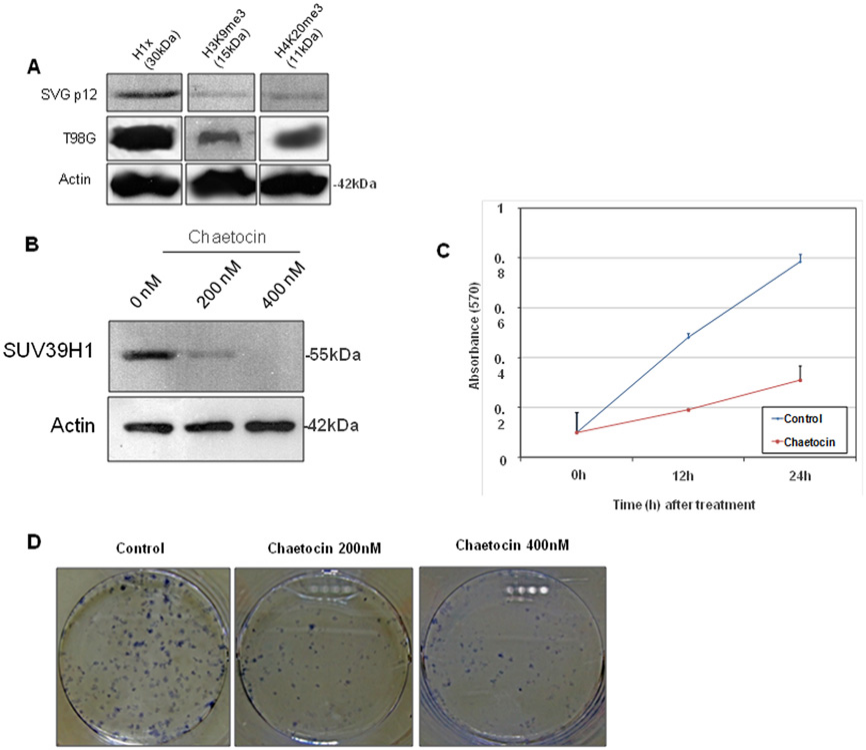
Western blot analysis of H1X, H3K9me3 and H4K20me3 expression levels in astroglial cells SVG p12 and glioma cell line T98G. (A). Inhibition of SUV39H1 protein levels after treatment with 200 nM and 400 nM chaetocin by Western blot (B). MTT proliferation assays performed in glioma cells at 12 h and 24 h following treatment with chaetocin, indicating reduced cell proliferation following suppression of SUV39H1. All values represent means ± standard deviation (SD) of four parallel wells (C). Clonogenic assays of T98G cells performed following 12h treatment with chaetocin (200nM, 400nM). Colony counts (%) were evaluated relative to untreated control for T98G chaetocin-treated cells. Colony counts were done in triplicate of three independents experiments. Both colony formation and migration of T98G cells was reduced following chaetocin treatment compared to controls.

The expression of histone H1x as well as the presence of histone modifications H3K9me3 and H4K20me3 was detected by western blot analysis in the astroglial cell line SVG p12 and glioma cell line T98G (Fig. 6A). In the former, the presence of H1x was more pronounced than H3K9me3 and H4K20me3.

In order to further examine the significance of SUV39H1 in gliomas progression, we inhibited its activity by using the chemical compound chaetocin. T98G glioma cells were treated with chaetocin (200 nM and 400 nM) for 12 and 24 hr. Twenty four hours after treatment, we verified inhibition of SUV39H1 by Western Blot analysis. Blotting for SUV39H1 indicated a partial reduction of SUV39H1 levels at 200 nM and complete inhibition at 400 nM at 24 hr post-treatment (Fig. 6B).

For the assessment of glioma cell proliferation after SUV39H1 inhibition, MTT assays were performed at 12h and 24h of culture. T98G cell proliferation in chaetocin-treated cells was significantly decreased compared to control (p < 0.05 at both time points (Fig. 6C).

### Suppression of SUV39H1 activity results in reduced clonogenic potential of glioma cells

T98G chaetocin-treated cells exhibited significantly reduced capacity to form colonies compared to untreated control cells (Fig. 6D). Specifically 200nM chaetocin-treated cells formed 31.8% colonies and 400nM chaetocin-treated cells formed only 18.7% colonies relative to control (p < 0.01).

### Suppression of SUV39H1 activity results in reduced migration of glioma cells

**Figure 7:**
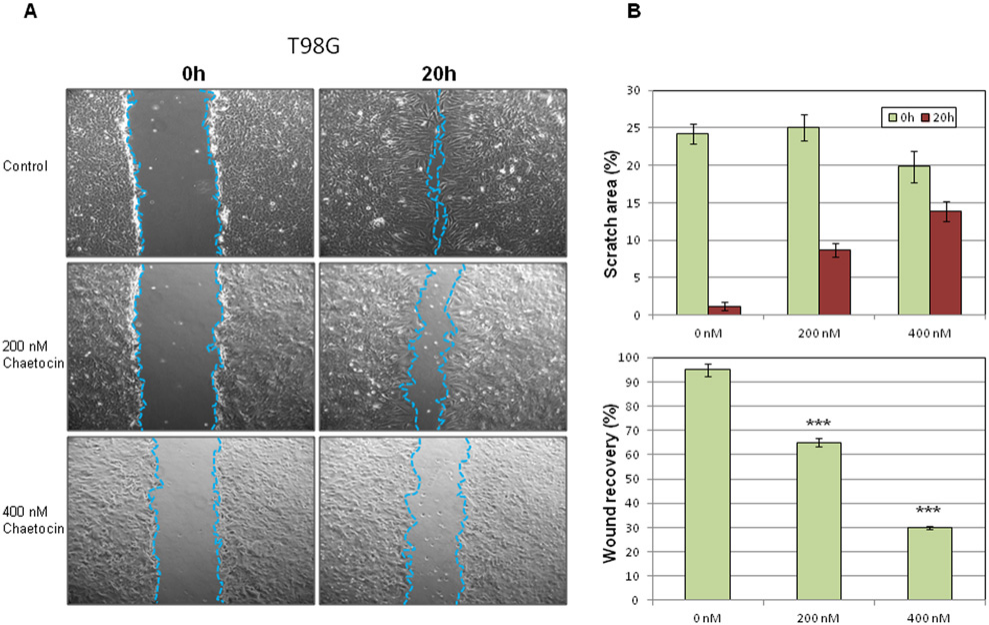
Monolayer scratch migration assays. **(A)** Monolayer scratch migration assays were performed following 200nM and 400nM chaetocin treatment for 20 h. (B) Cell migration in the scratch area was calculated for chaetocin-treated T98G cells. Wound recovery was also estimated for chaetocin-treated cells compared to untreated controls (***p < 0.001)

To investigate the effects of SUV39H1 on glioma cell migration ability, monolayer scratch migration assays were performed 20h after 200nM and 400nM chaetocin treatment (Fig. 7A). Cell migration in the scratch area was reduced by 64.8% and 29.6% in T98G chaetocin-treated cells (Fig. 7B). Wound recovery was 95.3% in untreated control, 64.7% in 200nM and 30.6% in 400nM T98G chaetocin-treated cells (p < 0.001 for 200nM and 400 nM chaetocin-treated cells compared to control, respectively, Fig. 7B). This data indicates the suppressive effect of SUV39H1 inhibition in the migratory capacity of glioma cells.

## Discussion

Accumulating evidence underlines the implication of epigenetic changes in glioma pathobiology, in terms of aberrant promoter methylation–induced gene silencing [23, 24, 25]. However, other mechanisms of epigenetic regulation, such as altered histone modification status and particularly disruption of the respective histone modifying enzymes are much less well characterized [2]. The present investigation was undertaken mainly in an attempt to shed light upon the clinical relevance of the two H3K9 methyltransferases-SETDB1 and SUV39H1 for which experimental data imply their involvement into gliomagenesis [19]. Two common histone modifications in gliomas, namely H3K9me3 and H4K20me3 were analysed in parallel [1, 12]. We also focused on linker histone H1x subtype, since we are largely ignorant of its potential role in gliomas.

The overall frequency of H3K9me3 positivity in our series was high, irrespective of astrocytic tumor grade, as reported by Venneti et al. [12]. Immunoreactivity was more pronounced in the adjacent normal brain compared to the neoplastic tissue, this difference resulting from the significantly lower levels in anaplastic astrocytomas and glioblastomas. Western blot analysis of glioma tissues of different grade as well as of normal brain tissue revealed a similar though less prominent expression pattern, probably due to the small number of samples examined. Analogous findings have been more recently reported in bladder cancer compared to normal urothelium [26]. The lower levels of H3K9me3 in astroglial tumors especially of higher grades, are most likely attributable to global DNA hypomethylation prevailing in high grade gliomas [27] as opposed to normal brain tissue [28]. Our observation is further corroborated by the recently reported enrichment of normal brain tissues with heterochromatin marks such as H3K9me2 and H3K27me3, whereas distinct euchromatin marks- AcH3, AcH4 and H3K4me2 were specifically detected in gliomas [29]. These differential histone signatures in normal brain and gliomas are in tune with the overexpression of the maintenance DNA methyltransferase (DNMT1) in the latter [29]. Whatever the actual mechanism of decreased H3K9me3 in gliomas grades 3 and 4 might be, our observation points towards this particular alteration being coincident with the late stages of gliomagenesis but is apparently at some variance with experimental findings according to which H3K9 trimethylation is thought to interfere with glial differentiation since neurosphere cultures overexpressing H3K9me3 display decreased GFAP protein. This effect, however, may be to some extent dependent upon the presence of *IDH1* mutation [30]. In addition, SETDB1 (KMT1E) mediated H3K9 methylation is reportedly required for the maintenance of embryonic stem cells by repressing trophectoderm differentiation [31].

We were unable to elicit any significant associations between H3K9me3 expression levels and the presence of IDH1R132H protein. Mutant *IDH1* is known to catalyse the production of the oncometabolite 2-hydroxyglutarate (2-HG) from a-ketoglutarate (a-KG) [32, 33]. 2-HG, being structurally similar to a-KG impairs the function of a-KG dependent dioxygenases, including the Jumonji C family of histone demethylases, thus accounting for the increased histone methylation marks in vitro [30, 34, 35]. In vivo, however, this relationship has been confirmed only in oligodendrogliomas and low-grade astrocytic tumors [12] and even in these cases a one-to-one correspondence has not been observed. Obviously, the small number of low-grade astrocytomas in our series is largely responsible for the disparity between the aforementioned study [12] and the present one. Notwithstanding, our findings underline the fact that in high-grade astrocytic tumors H3K9 is trimethylated by an as yet undefined *IDH1* mutant independent mechanism deregulating the complex dynamic balance between methyltransferases and demethylases. Alternatively, H3K9 trimethylation may be mediated by DNA methyltransferases [36, 37 38] of which DNMT1 and DNMT3 are known to be deregulated in glioblastoma [39, 40, 29].

Importantly, the expression of H3K9me3 bore no prognostic connotations either in the entire cohort or in glioblastomas. In contrast, H3K9 trimethylation reportedly confers a favorable prognosis in oligodendrogliomas [12]. It could be argued that a major reason for this difference might be the distinct mechanisms conducting H3K9 methylation in the two types of gliomas, i.e. *IDH1* mutation dependent in oligodendrogliomas versus *IDH1* mutation independent in high-grade astrocytic tumors, since *IDH1* mutations are advanced as one of the most powerful favorable outcome predictors in gliomas [41, 42].

A novel finding is that H3K9me3 levels increased in parallel with those of SETDB1 consistent with the notion that this histone mark is preferentially established by SETDB1 rather than by SUV39H1 in astrocytic tumors. It is, therefore, likely that these two HKMTs play distinct roles in the regulation of chromating remodeling in gliomas. In support of this notion, SETDB1 ablation in developing mouse brain caused a decrease in H3K9 trimethylation [16].

In the present investigation, we demonstrate for the first time that the expression of these two transcriptionally repressive HKMTs is up-regulated in astrocytic tumors with glioblastomas displaying elevated cytoplasmic SUV39H1 and SETDB1, but intriguingly lower nuclear SUV39H1. Although the significance of cytoplasmic SUV39H1 remains presently unknown, it has been recorded in oral squamous cell carcinoma [43] in which only nuclear SUV39H1 was positively associated with stage. Given that a negative correlation existed between nuclear and cytoplasmic SUV39H1, it could be hypothesized that this HKMT may invoke opposing functions depending on its subcellular localization. Taking into account the low level of expression in normal brain observed in the present study as well as in the one by Spyropoulou et al. [19] these findings speak in favor of SETDB1 and SUV39H1 being implicated in both the development and progression of astrocytic tumors. An elevated expression of SET domain HKMTs has been shown in immortalized bronchoepithelial cells [44] as well as with increasing breast cancer grade [45, 19]. In vitro experiments have most recently provided some insight into the biological significance of these two HKMTs in astrocytomas. Thus, their targeting by siRNA significantly reduced proliferation of the glioma cell lines GOS-3 by inducing apoptosis and reduced cell migration and clonogenic ability, SUV39H1 being more influential in this regard. On the other hand, SETDB1 down-regulation in a murine model resulted in severely impaired neurogenesis and astrocytic differentiation [16]. Further investigation of the potential functional role of SUV39H1 revealed a significant reduction of cell proliferation of T98G glioma cells upon treatment with the specific inhibitor chaetocin, followed by reduced colony formation and migration, strengthening the implication of SUV39H1 in glioma progression. It is worthy of note that knockdown of PRMT5-another methyltransferase targeting arginine residues on histone tails- had a similar effect on the clonogenic potential and viability of glioma cells [46].

A major finding emerging from the present investigation is the prognostic effect of SUV39H1 expression in the entire cohort, as well as in glioblastomas. As expected from SUV39H1 correlations with grade, nuclear SUV39H1 exerted a favorable effect as opposed to cytoplasmic SUV39H1. More importantly, the latter was documented only in the entire cohort implying that this effect is secondary to the positive association of cytoplasmic SUV39H1 with grade. SETDB1 appeared of lesser importance in this regard displaying a marginally significant favorable effect in univariate analysis in the entire cohort. These results are somewhat unexpected given the experimental data reported by Spyropoulou et al. [19] and rather assign a tumor suppressive role to nuclear SUV39H1 in astrocytic gliomas. Indeed, such an effect may be implied by the fact that double knockout of SUV39H1/H2 causes genomic instability, while SUV39H1 dependent senescence reportedly protects mice from Ras-driven invasive T-cell lymphoma [17]. On the contrary, Patani et al. [45] have shown that the expression profile of SUV39H1 is positively associated with tumor grade or TNM stage and inversely with disease-free survival in breast cancer. Most recently, expression of the protein arginine methyltransferase PRMT5 was also proposed as an adverse prognostic marker in glioblastoma [47]. Such controversial findings attest to the complexity of HKMTs, which may target multiple substrates yielding opposite effects dependent on the context.

We observed a gradual loss of H4K20me3 immunoexpression from grade 2, through grade 3 to grade 4. It is, therefore, reasonable to assume that depletion of this histone mark may be of importance for the progression of astrocytic tumors. In addition, the adjacent normal brain displayed significant levels of H4K20me3, lower from grade 2 but not significantly different from those of astrocytomas grades 3 and 4. Despite the association with grade, no relationship with survival was elicited. To the best of our knowledge, there is only one study investigating global H4K20 methylation levels in gliomas [1] in which no relationship with grade was documented but only with survival of secondary glioblastoma patients, being in overall agreement with our finding. The causes leading to altered H4K20me3 levels are not fully characterized since, to date, there is no known demethylase of H4K20, suggesting that H4K20 methylation is a stable mark. Of course, it seems likely that H4K20 demethylases will be discovered in the future as it has been done for H3 modifications [48]. Trimethylation of H4K20 leads to gene silencing by promoting the interaction with heterochromatin [14]. The loss of H4K20 trimethylation, which is considered a universal hallmark of neoplastic transformation, is presently assigned to the hypomethylation of DNA repetitive sequences- a well known characteristic of cancer cells in general and of glioblastoma cells in particular, linked to chromosomal instability [49, 50]. This loss of H4K20me3 appears to be cumulative during carcinogenesis. For example, it becomes more pronounced with progression from low- to high-grade lung neuroendocrine tumors [11] as in our study, as well as from low-grade to high-grade dysplasia of squamous epithelium in the lung [51]. H4K20 methylation decreases when cells are in G2/M [49, 52] which explains why neoplastic astrocytes in mitosis in our study were negative for H4K20me3. However, the increase in mitotic/proliferative activity from low- to high-grade astrocytomas may not be held entirely responsible for the corresponding decrease of H4K20me3 levels. This is because neoplastic cells have been shown to display lower H4K20me3 levels than normal cells even when they are both arrested in G0/G1 or G2/M phases [49]. The prognostic role of H4K20 methylation in tumors has been controversial with low levels having been predictive of favorable clinical outcome in myoinvasive bladder cancer [48] and of poor clinical outcome in stage I lung adenocarcinoma [51] and breast cancer [53]. These differences may reflect different expression profiles of histone modifying enzymes (i.e. KMT5A, KMT5B and KMT5C) in various tumor types [48].

Perhaps the most important findings of the present investigation relate to the expression pattern of H1x, a member of the linker histone family. Despite the importance of linker histones for the maintenance of higher order chromatin structure and for the regulation of gene expression, there is a striking paucity of information regarding the significance of H1 modifications in tumorigenesis. Interest in H1 has been tempered by its much lower level of evolutionary conservation compared to core histones leading to the assumption that linker histones are less relevant to chromatin biology [54]. We herein describe for the first time in a large series of astroglial tumors that the H1x variant of linker histone is expressed at high levels by the normal brain and significantly decreases during the transformation from low- to high- grade astrocytomas, this decrease constituting an adverse prognostic indicator. Most importantly, this favorable prognostic effect is maintained in multivariate analysis and is validated in an independent set of patients. Another subtype of H1–H1.0 extracted from glioma tissues was also shown to portend a favorable outcome, although this finding was based on the analysis of only 29 patients [20]. These findings suggest that in high-grade astrocytomas the higher order chromatin structure is impaired. Our findings come in broad agreement with those recently reported in ovarian epithelial tumors in which H1x (along with H1.1, H1.4 and H1a) mRNA levels were significantly reduced in adenocarcinomas compared with adenomas [55]. It has been postulated that the degree of chromatin compaction resulting from alterations in the relative levels of specific H1 subtypes impacts on DNA damage response, cell migration and invasiveness [56, 57], although this effect is multifaceted and context/H1 subtype dependent [55].

It is worthy of note that our study reveals a positive correlation of H1x expression with H4K20me3 and H3K9me3 suggesting that alterations in the linker histone levels potentially contribute to maintenance or establishment of specific methylation patterns in histones H3 and H4. Although there is no relevant observation in tumor context, studies in mouse embryonic stem cells revealed a reduction in global H3K27 methylation levels when H1 expression is lowered by 50% [58]. Furthermore, in experiments with reconstitutive nucleosomes and enzyme complexes, H1 has been found to affect H3 methylation activity by determining the substrate preference of EZH2 histone methyltransferase complex [59]. Our findings should thus inspire the investigators to explore the significance of the remaining subtypes of H1 in astrocytic tumors and prompt future experimentation to dissect and verify the contribution of H1 alterations in gliomagenesis.

In conclusion, the present investigation stands in favor of the epigenetic phenotype in terms of decreased H4 trimethylation at Lys20 being involved in the progression of diffuse astrocytic tumors. Although H3K9 trimethylation appears of lesser biologic relevance in this regard, our findings highlight the implication of its corresponding methyltransferases SUV39H1 and SETDB1 in the development as well as the acquisition of a more aggressive phenotype as a function of their correlation with grade and, as far as SUV39H1 is concerned, with survival. Notably, SUV39H1 correlations with survival and grade are both guided by its subcellular localization. More importantly, deregulation of H1x subtype is brought forward as a mechanism assisting the late steps of gliomagenesis and as a prognostic biomarker of favorable significance independent of classical prognosticators. This novel finding warrants the elucidation of the underlining machinery and the potential involvement of other H1 subtypes in the pathobiology of astrocytic tumors.

## References

[1] Liu BL, Cheng JX, Zhang X, Wang R, Zhang W, et al (2010) Global histone modification patterns as prognostic markers to classify glioma patients. Cancer Epidemiol Biomarkers Prev. 19: 888–96. 10.1158/1055-9965.EPI-10-0454 20978174

[2] Spyropoulou A, Piperi C, Adamopoulos C, Papavassiliou AG. (2013) Deregulated chromatin remodeling in the pathobiology of brain tumors. Neuromolecular Med. 2013 15: 1–24. 10.1007/s12017-012-8205-y 23114751

[3] Holliday R. (1987) The inheritance of epigenetic defects. Science. 238: 163–70. 10.1126/science.3310230 3310230

[4] Luger K. (2006) Dynamic nucleosomes. Chromosome Res. 14: 5–16. 10.1007/s10577-005-1026-1 16506092

[5] Fischle W, Wang Y, Allis CD. (2003) Histone and chromatin cross-talk. *Cu*rr Opin Cell Biol. 15: 172–83. 10.1016/S0955-0674(03)00013-9 12648673

[6] Luger K, Mäder AW, Richmond RK, Sargent DF, Richmond TJ. (1997) Crystal structure of the nucleosome core particle at 2.8 A resolution. Nature. 389: 251–60. 10.1038/38444 9305837

[7] Kornberg RD, Lorch Y. (1999) Twenty-five years of the nucleosome, fundamental particle of the eukaryote chromosome. Cell. 98: 285–94. 10.1016/S0092-8674(00)81958-3 10458604

[8] Harvey AC, Downs JA. What functions do linker histones provide? (2004) Mol Microbiol. 53: 771–5. 10.1111/j.1365-2958.2004.04195.x 15255891

[9] McBryant SJ, Lu X, Hansen JC. (2010) Multifunctionality of the linker histones: an emerging role for protein-protein interactions. Cell Res. 20: 519–28. 10.1038/cr.2010.35 20309017 PMC2919278

[10] Berger SL. The complex language of chromatin regulation during transcription. (2007) Nature. 447: 407–12. 10.1038/nature05915 17522673

[11] Li F, Ye B, Hong L, Xu H, Fishbein MC. (2011) Epigenetic modifications of histone h4 in lung neuroendocrine tumors. Appl Immunohistochem Mol Morphol. 19: 389–94. 10.1097/PAI.0b013e3182108e2e 21415707

[12] Venneti S, Felicella MM, Coyne T, Phillips JJ, Gorovets D, et al (2013) Histone 3 lysine 9 trimethylation is differentially associated with isocitrate dehydrogenase mutations in oligodendrogliomas and high-grade astrocytomas. J Neuropathol Exp Neurol. 72: 298–306. 10.1097/NEN.0b013e3182898113 23481705 PMC3615673

[13] Chi P, Allis CD, Wang GG. Covalent histone modifications--miswritten, misinterpreted and mis-erased in human cancers. (2010) Nat Rev Cancer. 10: 457–69. 10.1038/nrc2876 20574448 PMC3262678

[14] Chernov AV, Sounni NE, Remacle AG, Strongin AY. (2009) Epigenetic control of the invasion-promoting MT1-MMP/MMP-2/TIMP-2 axis in cancer cells. J Biol Chem. 284: 12727–34. 10.1074/jbc.M900273200 19286653 PMC2676002

[15] Sarraf SA, Stancheva I. (2004) Methyl-CpG binding protein MBD1 couples histone H3 methylation at lysine 9 by SETDB1 to DNA replication and chromatin assembly. Mol Cell. 15: 595–605. 10.1016/j.molcel.2004.06.043 15327775

[16] Tan SL, Nishi M, Ohtsuka T, Matsui T, Takemoto K, et al (2012) Essential roles of the histone methyltransferase ESET in the epigenetic control of neural progenitor cells during development. Development. 139: 3806–16. 10.1242/dev.082198 22991445

[17] He Y, Korboukh I, Jin J, Huang J. (2012) Targeting protein lysine methylation and demethylation in cancers. Acta Biochim Biophys Sin (Shanghai). 44: 70–9. 10.1093/abbs/gmr109 22194015 PMC3244655

[18] Isham CR, Tibodeau JD, Jin W, Xu R, Timm MM et al (2007) Chaetocin: a promising new antimyeloma agent with in vitro and in vivo activity mediated via imposition of oxidative stress. Blood. 109: 2579–88. 10.1182/blood-2006-07-027326 17090648 PMC1852204

[19] Spyropoulou A, Gargalionis A, Dalagiorgou G, Adamopoulos C, Papavassiliou KA et al (2014) Role of Histone Lysine Methyltransferases SUV39H1 and SETDB1 in Gliomagenesis: Modulation of Cell Proliferation, Migration, and Colony Formation. Neuromolecular Med. 16: 70–82. 10.1007/s12017-013-8254-x 23943221

[20] Gabrovsky N, Georgieva M, Laleva M, Uzunov K, Miloshev G. (2013) Histone H1.0—a potential molecular marker with prognostic value for patients with malignant gliomas. Acta Neurochir (Wien). 155: 1437–42. 10.1007/s00701-013-1802-1 23812966

[21] Louis DN, Ohgaki H, Wiestler OD, Cavenee WK (2007) The 2007 WHO classification of tumors of the central nervous system. Lyon: IARC. 10.1007/s00401-007-0243-4 PMC1929165 17618441

[22] Lachin JM, Foulkes MA. (1986) Evaluation of sample size and power for analyses of survival with allowance for nonuniform patient entry, losses to follow-up, noncompliance, and stratification. Biometrics. 42: 507–19. 10.2307/2531201 3567285

[23] Esteller M. (2007) Cancer epigenomics: DNA methylomes and histone-modification maps. Nat Rev Genet. 8: 286–98. 10.1038/nrg2005 17339880

[24] Esteller M. (2008) Epigenetics in cancer. N Engl J Med. 358: 1148–59. 10.1056/NEJMra072067 18337604

[25] Seligson DB, Horvath S, McBrian MA, Mah V, Yu H et al (2009) Global levels of histone modifications predict prognosis in different cancers. Am J Pathol. 174: 1619–28. 10.2353/ajpath.2009.080874 19349354 PMC2671251

[26] Ellinger J, Bachmann A, Göke F, Behbahani TE, Baumann C et al (2014) Alterations of Global Histone H3K9 and H3K27 Methylation Levels in Bladder Cancer. 93:113–8. 10.1159/000355467 24556868

[27] Wang H, Feng Y, Bao Z, Jiang C, Yan W et al (2013) Epigenetic silencing of KAZALD1 confers a better prognosis and is associated with malignant transformation/progression in glioma. Oncol Rep. 30: 2089–96. 24002581 10.3892/or.2013.2706

[28] Saratsis AM, Kambhampati M, Snyder K, Yadavilli S, Devaney JM et al (2013) Comparative multidimensional molecular analyses of pediatric diffuse intrinsic pontine glioma reveals distinct molecular subtypes. Acta Neuropathol. 10.1007/s00401-013-1218-2 24297113 PMC4028366

[29] Rajendran G, Shanmuganandam K, Bendre A, Muzumdar D, Goel A et al (2011) Epigenetic regulation of DNA methyltransferases: DNMT1 and DNMT3B in gliomas. J Neurooncol. 104: 483–94. 10.1007/s11060-011-0543-3 21229291

[30] Lu C, Ward PS, Kapoor GS, Rohle D, Turcan S et al (2012) IDH mutation impairs histone demethylation and results in a block to cell differentiation. Nature. 483: 474–8. 10.1038/nature10860 22343901 PMC3478770

[31] Lohmann F, Loureiro J, Su H, Fang Q, Lei H et al (2010) KMT1E mediated H3K9 methylation is required for the maintenance of embryonic stem cells by repressing trophectoderm differentiation. Stem Cells. 28: 201–12. 20014010 10.1002/stem.278

[32] Dang L, White DW, Gross S, Bennett BD, Bittinger MA et al (2010) Cancer-associated IDH1 mutations produce 2-hydroxyglutarate. Nature. 465: 966 10.1038/nature09132 20559394 PMC3766976

[33] Ward PS, Patel J, Wise DR, Abdel-Wahab O, Bennett BD et al (2010) The common feature of leukaemia-associated IDH1 and IDH2 mutations is a neomorphic enzyme activity converting alpha-ketoglutarate to 2-hydroxyglutarate. Cancer Cell. 17: 225–34. 10.1016/j.ccr.2010.01.020 20171147 PMC2849316

[34] Chowdhury R, Yeoh KK, Tian YM, Hillringhaus L, Bagg EA et al (2011) The oncometabolite 2-hydroxyglutarate inhibits histone lysine demethylases. EMBO Rep. 12: 463–9. 10.1038/embor.2011.43 21460794 PMC3090014

[35] Xu W, Yang H, Liu Y, Yang Y, Wang P et al (2011) Oncometabolite 2-hydroxyglutarate is a competitive inhibitor of α-ketoglutarate-dependent dioxygenases. Cancer Cell. 18,19:17–30. 10.1016/j.ccr.2010.12.014 21251613 PMC3229304

[36] Rai K, Nadauld LD, Chidester S, Manos EJ, James SR et al (2006) Zebra fish Dnmt1 and Suv39h1 regulate organ-specific terminal differentiation during development. Mol Cell Biol. 26: 7077–85. 10.1128/MCB.00312-06 16980612 PMC1592902

[37] Estève PO, Chin HG, Smallwood A, Feehery GR, Gangisetty O et al (2006) Direct interaction between DNMT1 and G9a coordinates DNA and histone methylation during replication. Genes Dev. 20: 3089–103. 10.1101/gad.1463706 17085482 PMC1635145

[38] Rai K, Jafri IF, Chidester S, James SR, Karpf AR et al (2010) Dnmt3 and G9a cooperate for tissue-specific development in zebrafish. J Biol Chem. 285: 4110–21. 10.1074/jbc.M109.073676 19946145 PMC2823551

[39] Fanelli M, Caprodossi S, Ricci-Vitiani L, Porcellini A, Tomassoni-Ardori F et al (2008) Loss of pericentromeric DNA methylation pattern in human glioblastoma is associated with altered DNA methyltransferases expression and involves the stem cell compartment. Oncogene. 27: 358–65. 10.1038/sj.onc.1210642 17653095

[40] Kreth S, Thon N, Eigenbrod S, Lutz J, Ledderose C et al (2011) O-methylguanine-DNA methyltransferase (MGMT) mRNA expression predicts outcome in malignant glioma independent of MGMT promoter methylation. PLoS One. 6: e17156 10.1371/journal.pone.0017156 21365007 PMC3041820

[41] Metellus P, Coulibaly B, Colin C, de Paula AM, Vasiljevic A et al (2010) Absence of IDH mutation identifies a novel radiologic and molecular subtype of WHO grade 2 gliomas with dismal prognosis. Acta Neuropathol. 120: 719–29. 10.1007/s00401-010-0777-8 21080178

[42] Myung JK, Cho HJ, Park CK, Kim SK, Phi JH et al (2012) IDH1 mutation of gliomas with long-term survival analysis. Oncol Rep. 28: 1639–44. 22922798 10.3892/or.2012.1994

[43] Chen JH, Yeh KT, Yang YM, Chang JG, Lee HE et al (2013) High expressions of histone methylation- and phosphorylation-related proteins are associated with prognosis of oral squamous cell carcinoma in male population of Taiwan. Med Oncol. 30: 513 10.1007/s12032-013-0513-z 23504335

[44] Watanabe H, Soejima K, Yasuda H, Kawada I, Nakachi I et al (2008) Deregulation of histone lysine methyltransferases contributes to oncogenic transformation of human bronchoepithelial cells. Cancer Cell Int. 8, 15 10.1186/1475-2867-8-15 18980680 PMC2584620

[45] Patani N, Jiang WG, Newbold RF, Mokbel K. (2011) Histone-modifier gene expression profiles are associated with pathological and clinical outcomes in human breast cancer. Anticancer Res. 31: 4115–25. 22199269

[46] Han X, Li R, Zhang W, Yang X, Wheeler CG et al (2014) Expression of PRMT5 correlates with malignant grade in gliomas and plays a pivotal role in tumor growth in vitro. J Neurooncol. 118:61–72. 10.1007/s11060-014-1419-0 24664369 PMC4076054

[47] Yan F, Alinari L, Lustberg ME, Martin LK, Cordero-Nieves HM et al (2014) Genetic validation of the protein arginine methyltransferase PRMT5 as a candidate therapeutic target in glioblastoma. Cancer Re. 15,74:1752–65. 10.1158/0008-5472.CAN-13-0884 24453002 PMC3959215

[48] Schneider AC, Heukamp LC, Rogenhofer S, Fechner G, Bastian PJ et al (2011) Global histone H4K20 trimethylation predicts cancer-specific survival in patients with muscle-invasive bladder cancer. BJU Int. 108: E290–6. 10.1111/j.1464-410X.2011.10203.x 21627749

[49] Fraga MF, Ballestar E, Villar-Garea A, Boix-Chornet M, Espada J et al (2005) Loss of acetylation at Lys16 and trimethylation at Lys20 of histone H4 is a common hallmark of human cancer. Nat Genet. 37: 391–400. 10.1038/ng1531 15765097

[50] Martinez R, Esteller M. (2010) The DNA methylome of glioblastoma multiforme. Neurobiol Dis. 39: 40–6. 10.1016/j.nbd.2009.12.030 20064612

[51] Van Den Broeck A, Brambilla E, Moro-Sibilot D, Lantuejoul S, Brambilla C et al (2008) Loss of histone H4K20 trimethylation occurs in preneoplasia and influences prognosis of non-small cell lung cancer. Clin Cancer Res. 14: 7237–45. 10.1158/1078-0432.CCR-08-0869 18974389

[52] Sarg B, Koutzamani E, Helliger W, Rundquist I, Lindner HH. (2002) Postsynthetic trimethylation of histone H4 at lysine 20 in mammalian tissues is associated with aging. J Biol Chem. 277: 39195–201. 10.1074/jbc.M205166200 12154089

[53] Elsheikh SE, Green AR, Rakha EA, Powe DG, Ahmed RA et al (2009) Global histone modifications in breast cancer correlate with tumor phenotypes, prognostic factors, and patient outcome. Cancer Res. 69: 3802–9. 10.1158/0008-5472.CAN-08-3907 19366799

[54] Harshman SW, Young NL, Parthun MR, Freitas MA. (2013) H1 histones: current perspectives and challenges. Nucleic Acids Res. 41: 9593–609. 10.1093/nar/gkt700 23945933 PMC3834806

[55] Medrzycki M, Zhang Y, McDonald JF, Fan Y. (2012) Profiling of linker histone variants in ovarian cancer. Front Biosci. 17, 396–406. 10.2741/3934 22201751 PMC3754803

[56] Murga M, Jaco I, Fan Y, Soria R, Martinez-Pastor B et al (2007) Global chromatin compaction limits the strength of the DNA damage response. J Cell Biol. 178: 1101–8. 10.1083/jcb.200704140 17893239 PMC2064646

[57] Gerlitz G, Bustin M. (2010) Efficient cell migration requires global chromatin condensation. J Cell Sci. 123: 2207–17. 10.1242/jcs.058271 20530575 PMC2886743

[58] Fan Y, Nikitina T, Zhao J, Fleury TJ, Bhattacharyya R et al (2005) Histone H1 depletion in mammals alters global chromatin structure but causes specific changes in gene regulation. Cell. 123: 1199–212. 10.1016/j.cell.2005.10.028 16377562

[59] Martin C, Cao R, Zhang Y. (2006) Substrate preferences of the EZH2 histone methyltransferase complex. J Biol Chem. 281: 8365–70. 10.1074/jbc.M513425200 16431907

